# Dr. A. Rostang

**Published:** 1842-12

**Authors:** 


***Dr. A. Rostang, Dentist, of Cincinnati.***—We understand that this gentleman is about to visit Paris, for the purpose of locating himself in that metropolis. We have heard Dr. R. spoken of as a skilful practitioner, and in leaving America for France, he will take with him, the best wishes of many friends for his success in that country. Dr. Rostang enjoys a high reputation as a surgeon dentist in Cincinnati, and his leaving there, will be regretted by very many.

